# Epitope Sequences in Dengue Virus NS1 Protein Identified by Monoclonal Antibodies

**DOI:** 10.3390/antib6040014

**Published:** 2017-10-15

**Authors:** Leticia Barboza Rocha, Rubens Prince dos Santos Alves, Bruna Alves Caetano, Lennon Ramos Pereira, Thais Mitsunari, Jaime Henrique Amorim, Juliana Moutinho Polatto, Viviane Fongaro Botosso, Neuza Maria Frazatti Gallina, Ricardo Palacios, Alexander Roberto Precioso, Celso Francisco Hernandes Granato, Danielle Bruna Leal Oliveira, Vanessa Barbosa da Silveira, Daniela Luz, Luís Carlos de Souza Ferreira, Roxane Maria Fontes Piazza

**Affiliations:** 1 Laboratório de Bacteriologia, Instituto Butantan, São Paulo, 05503-900 SP, Brazil; leticia.rocha@butantan.gov.br (L.B.R.); bruna.caetano@butantan.gov.br (B.A.C.); thais.mitsunari@butantan.gov.br (T.M.); juliana.yassuda@butantan.gov.br (J.M.P.); daniedaluz@yahoo.com.br (D.L.); 2 Laboratório de Desenvolvimento de Vacinas, Instituto de Ciências Biomédicas, Universidade de São Paulo, São Paulo, 05508-000, SP, Brazil; rubens.bmc@gmail.com (R.P.d.S.A.); lennon_rp@usp.br (L.R.P.); jh.biomedico@gmail.com (J.H.A.); lcsf@usp.br (L.C.d.S.F.); 3 Laboratório de Virologia, Instituto Butantan, São Paulo, 05503-900 SP, Brazil; viviane.botosso@butantan.gov.br; 4 Divisão de Desenvolvimento Tecnológico e Produção; Instituto Butantan, São Paulo, 05503-900, SP, Brazil; neuza.gallina@butantan.gov.br; 5 Divisão de Ensaios Clínicos e Farmacovigilância, Instituto Butantan, São Paulo, 05503-900, SP, Brazil; ricardo.palacios@butantan.gov.br (R.P.); alexander.precioso@butantan.gov.br (A.R.P.); 6 Departamento de Medicina, Disciplina de Doenças Infecciosas e Parasitárias, Universidade Federal de São Paulo, São Paulo, 04023-062, SP, Brazil; celso.granato@grupofleury.com.br; 7 Laboratório de Virologia Molecular e Clínica, Departamento de Microbiologia, Instituto de Ciências Biomédicas, Universidade de São Paulo, São Paulo, 05508-000, SP, Brazil; danibruna@gmail.com (D.B.L.O.); vanessa.silveirabio@gmail.com (V.B.d.S.)

**Keywords:** dengue virus, NS1, Zika virus, mAbs, antibody recognition, amino acid sequences

## Abstract

Dengue nonstructural protein 1 (NS1) is a multi-functional glycoprotein with essential functions both in viral replication and modulation of host innate immune responses. NS1 has been established as a good surrogate marker for infection. In the present study, we generated four anti-NS1 monoclonal antibodies against recombinant NS1 protein from dengue virus serotype 2 (DENV2), which were used to map three NS1 epitopes. The sequence ^193^AVHADMGYWIESALNDT^209^ was recognized by monoclonal antibodies 2H5 and 4H1BC, which also cross-reacted with Zika virus (ZIKV) protein. On the other hand, the sequence ^25^VHTWTEQYKFQPES^38^ was recognized by mAb 4F6 that did not cross react with ZIKV. Lastly, a previously unidentified DENV2 NS1-specific epitope, represented by the sequence ^127^ELHNQTFLIDGPETAEC^143^, is described in the present study after reaction with mAb 4H2, which also did not cross react with ZIKV. The selection and characterization of the epitope, specificity of anti-NS1 mAbs, may contribute to the development of diagnostic tools able to differentiate DENV and ZIKV infections.

## 1. Introduction

Dengue fever is an important mosquito-borne and the most prevalent and costly arbovirus affecting humans, caused by one of the four serotypes of dengue virus (DENV 1–4) [1]. In the last decade, a large number of dengue epidemics have occurred, which resulted in enormous economic and human loss in parts of Asia and South America [2,3]. Considering Brazil only, more than three million cases of confirmed dengue infections occurred between 2015 and 2017, with 70 cases per 100,000 inhabitants [4].

The DENV genome is composed of a single positive-sense RNA that encodes a single viral polyprotein that is further processed by viral and host proteases into three structural proteins (C, prM/M, and E) and seven nonstructural proteins (NS1, NS2A, NS2B, NS3, NS4A, NS4B, and NS5). NS1 is the first nonstructural protein to be translated and is essential to virus replication [5]. It is a conserved N-linked glycoprotein with a variable molecular mass of 46–55 kDa, which depends on its glycosylation status [6]. The NS1 protein can be found as a dimer associated with vesicular compartments within the cell, where it plays an important role as an essential cofactor in the virus replication process [7]. Alternatively, NS1 can be secreted into the extracellular space as a hexameric lipoprotein particle [8] that interacts with several plasma proteins [9,10].

The recent introduction of the Zika virus (ZIKV) to the American continent represented a regional and worldwide public health challenge [11]. The close evolutionary relationship between DENV and ZIKV is reflected by the high sequence conservation of both structural and non-structural proteins [12]. In this aspect, the identification of monoclonal antibodies (mAbs) able to react specifically with DENV or cross-react with ZIKV proteins is a relevant feature for the validation of the diagnostic tools based on the NS1 protein.

In pioneering work by Falconar et al. [8], the immunogenic regions of DENV2 NS1 employing mAbs were extensively studied. Recently, certain studies have been using new methods to predict the binding epitopes of proteins to specific antibodies [13,14]. This approach was also applied to identify binding epitopes of DENV NS1 protein serotypes [15,16,17]. Also, the crystal structure of the DENV2 NS1 protein (PDB code: 4O6B) has been solved in both dimeric and hexameric configurations [6], which provides a useful guide for the selection of potential epitopes for therapy and vaccine strategies.

In the present study, recombinant DENV2 NS1 was used to immunize mice and generate murine mAbs. Four mAbs were isolated, purified, characterized and tested for reactivity with native NS1 produced by all DENV serotypes in Vero-infected cells and also for cross-reactivity with ZIKV NS1.

## 2. Results

### 2.1. Isolation and Characterization of NS1-Specific DENV mAbs

Fusion of popliteal lymph node cells, from mice immunized with DENV2 rNS1, with a non-Ig-secreting or synthesizing line derived from a cell line created by fusing a BALB/c mouse spleen cell and the mouse myeloma P3X63Ag8 (SP2/O-Ag14) mouse myeloma cells, generated 25 secretory hybridomas. Among them, four hybridomas were selected by enzyme-linked immunosorbent assay (ELISA) and sub cloned by limiting dilution and named as 4F6, 4H2, 4H1BC, and 2H5. The clones were expanded, supernatants collected and mAbs purified for further characterization. Accordingly, mAbs 4F6 and 4H2 were characterized as IgG2a (immunoglobulin G), and 2H5 and 4H1BC as IgG1. The affinity constants were similar (10^−8^ M) as well as their reactivity with and limits of detection of NS1 (Table 1).

**Figure 1:**
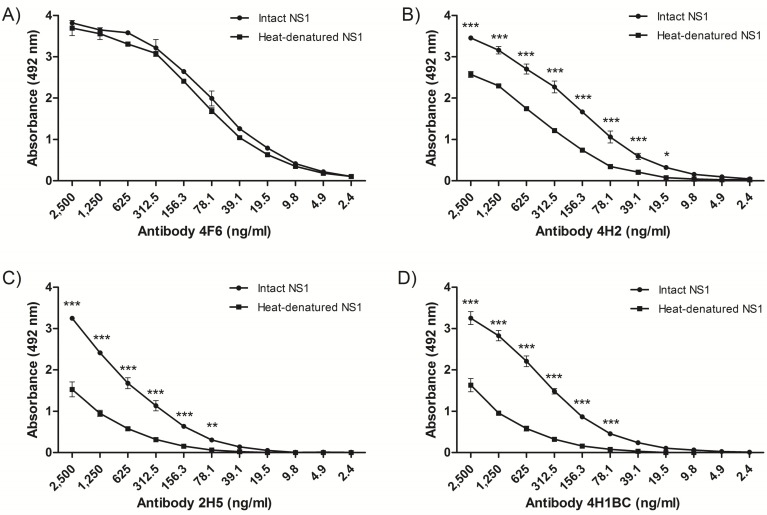
Characterization of nonstructural protein 1-specific (NS1) monoclonal antibodies (mAbs) reactivity by enzyme-linked immunosorbent assay (ELISA). Reactivity of mAbs to heated-treated or intact rNS1, as solid phase-bound antigens. The mAbs 4F6 (**A**), 4H2 (**B**), 2H5 (**C**) and 4H1BC (**D**) were serially diluted (log2) from an initial concentration of 2.5 µg/mL. Each well was adsorbed with 400 ng of rNS1. Heat denaturation was performed at 100 °C for 10 min. Statistical analyses were performed by two-way variance analysis followed by Bonferroni’s post-test. (*** *p* < 0.01; ** *p* < 0.1; * *p* < 0.5).

The recognition pattern of the four NS1 mAbs was evaluated by ELISA using either intact or heat-denaturated rNS1. All NS1 mAbs recognized the intact rNS1 protein, and although mAb 4F6 reacted similarly with the intact and the heated-treated rNS1 (Figure 1A), the other three mAbs (4H2, 2H5 and 4H1BC) reacted more efficiently with the intact protein (Figure 1B–D, respectively), which indicated that the recognized epitopes were, at least, partially represented by conformational structures. All four MAbs also recognized rNS1 in an immunoblot assay (Figure S1).

### 2.2. Detection of Native DENV2 NS1 and Epitope Mapping

**Figure 2:**
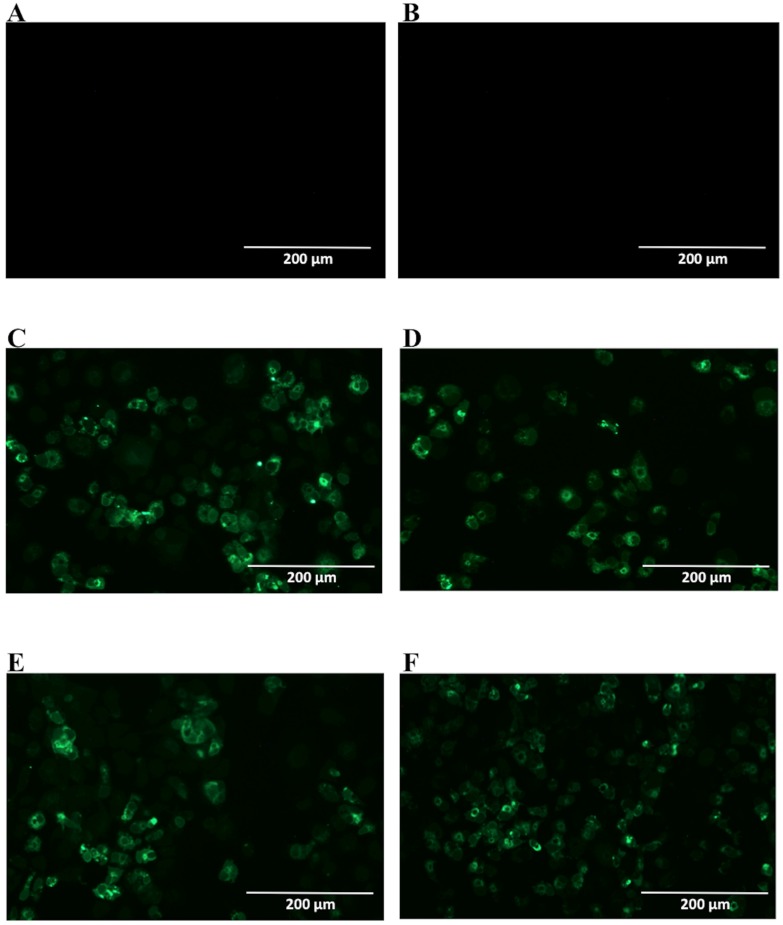
Reactivity of NS1-specific mAbs to dengue-serotype 2-infected Vero cells. Cells were infected with a multiplicity of infection (MOI) of 0.5, fixed, permeabilized and treated with each of the tested mAbs 48 h post infection. Then, cells were labeled with Alexa fluor^®^ conjugated goat-anti mouse IgG. The negative controls: Mock-infected cells treated with a pool of mAbs anti-NS1 (**A**) and DENV2-infected cells labeled only with secondary antibody (**B**); Tested mAbs: (**C**) 4F6; (**D**) 4H2; (**E**) 2H5 and (**F**) 4H1BC. Magnification of 200×.

**Figure 3:**
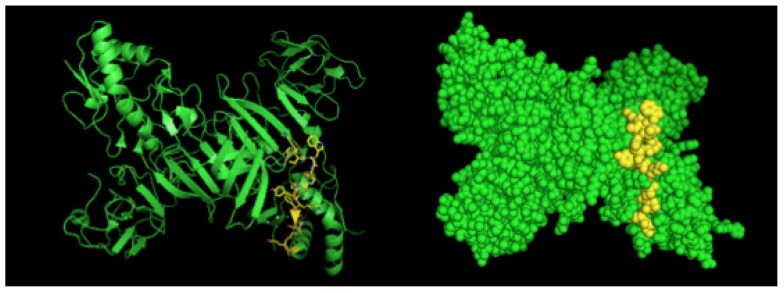
Three-dimensional structural model of a NS1 dimer and regions corresponding to epitopes recognized by 4F6 mAb. The NS1 3D model was generated by the program Python Molecular (PyMOL) in green. The sequence ^25^VHTWTEQYKFQPES^38^ is highlighted in yellow.

**Figure 4:**
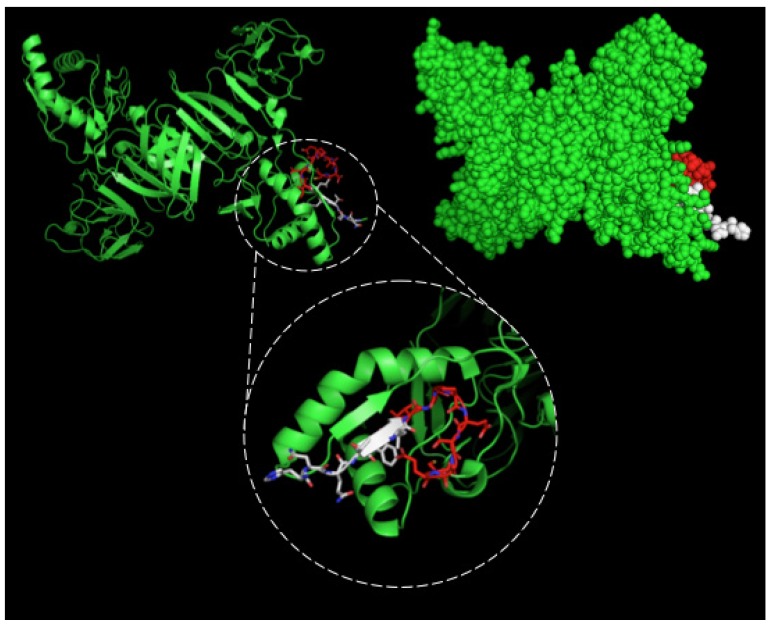
Three-dimensional structural model of a NS1 dimer and regions corresponding to epitopes recognized by 4H2 mAb. The NS1 3D model was generated by the program PyMOL in green. The sequence ^127^ELHNQTFLIDGPETAEC^143^ is highlighted in red and white. In the detail the red structure represents the novel nine-amino acid sequence described herein.

**Figure 5:**
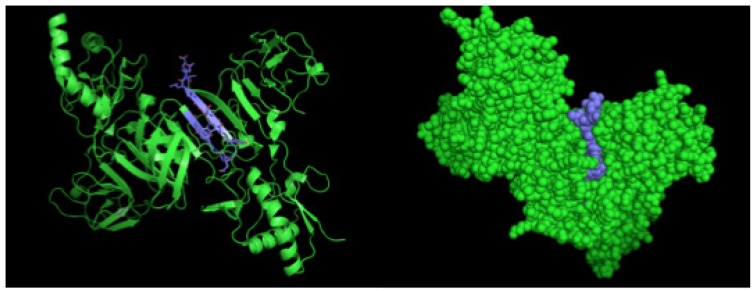
Three-dimensional structural model of a NS1 dimer and regions corresponding to epitopes recognized by 2H5 and 4H1BC mAbs. The NS1 3D model was generated by the program PyMOL in green. The sequence ^193^AVHADMGYWIESALNDT^209^ is highlighted in blue.

After selection, mAbs were tested by immunofluorescence assays using fixed DENV2-infected Vero cells. All four mAbs recognized the native viral NS1 expressed in infected cells, as shown in Figure 2. To localize the specific mAbs binding sites/epitopes, peptide mapping array experiments were performed (Figure S2). The results showed that mAb 4F6 reacted with the peptide corresponding to the sequence ^25^VHTWTEQYKFQPES^38^ of NS1 (Table 1), which is located in an external loop of the protein 3D structure (Figure 3). The 4H2 mAb recognized the peptide corresponding to the sequence ^127^ELHNQTFLIDGPETAEC^143^ of NS1 (Table 1), which is located in beta-sheets in an external region of the protein 3D structure (Figure 4). The other two mAbs (2H5 and 4H1BC) showed the same binding specificity and recognized the peptide ^193^AVHADMGYWIESALNDT^209^ (Table 1). This sequence was also located in a beta-sheet structure, located in an internal region of the protein (Figure 5). The analysis of epitope conservancy in several strains of DENV serotypes as well as Zika strains is detailed in Table S1.

### 2.3. Analyses of mAbs’ Cross-Reactivity with Different DENV Serotype and ZIKV

**Figure 6:**
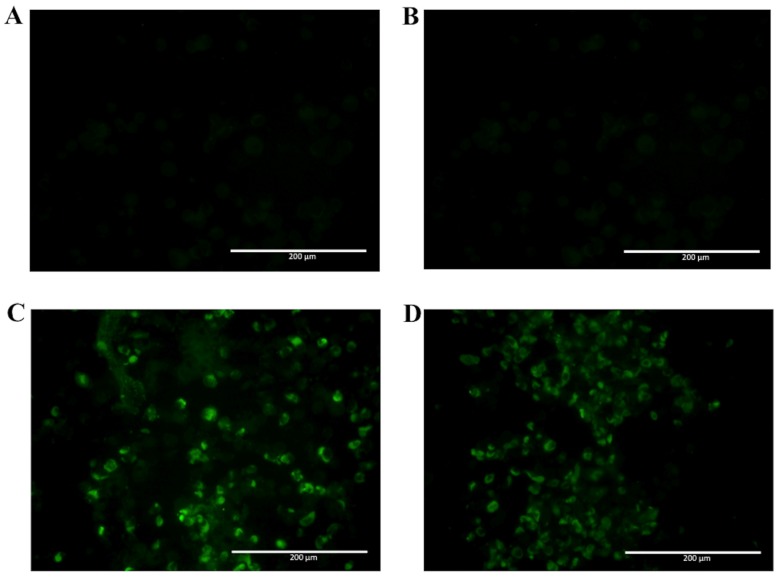
Reactivity of NS1-specific mAbs to zika virus-infected Vero cells. Cells were infected with a MOI of 0.05. 72 h post infection, cells were fixed, permeabilized and treated with each of the tested mAbs. Then, cells were labeled with FITC-conjugated goat-anti mouse IgG. Tested mAbs: (**A**) 4F6; (**B**) 4H2; (**C**) 2H5 and (**D**) 4H1BC. Magnification of 200×.

**Figure 7:**
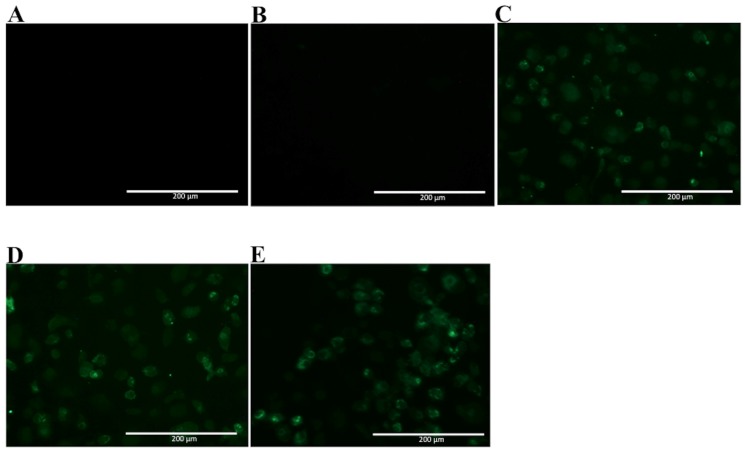
Reactivity of 4H2 mAb to Vero cells infected with DENV of different serotypes. Vero cells were infected with a MOI of 0.5, fixed, permeabilized and treated with mAb 4H2 48 h post infection. Then, cells were labeled with Alexa fluor^®^ conjugated goat-anti mouse IgG. The negative controls: (**A**) Mock infected cells treated with a pool of mAbs anti-NS1 and (**B**) DENV-infected cells labeled only with secondary antibody; Tested DENV serotypes: (**C**) DENV1; (**D**) DENV3 and (**E**) DENV4. Magnification of 200×.

Since NS1 shares a high homology with amino acid sequences found among different flavivirus, the selected mAbs were tested for recognition of native ZIKV NS1 by immunofluorescence assay, using fixed ZIKV-infected Vero cells. In Figure 6, two mAbs are observed to cross-react with native ZIKV-NS1 in this test (2H5 and 4H1BC) (Figure 6C,D, Table 1). The other two mAbs (4F6 and 4H2) were specific for DENV NS1 (Figure 6A,B, Table 1). We also tested in vitro the reactivity of 4F6 and 4H2 mAbs with DENV serotypes, other than DENV2, and only the 4H2 mAb reacted with all four DENV serotypes (Figure 7, Table 1).

## 3. Discussion

The DENV NS1 has been used as a target antigen against dengue infection either for vaccines, antiviral drug design or diagnostic methods. Indeed, this protein is secreted by infected cells during the acute phase and circulates in the blood at high concentrations [19]. Nevertheless, the NS1 shares parts of its amino acid sequence among flavivirus. In the present study, we generated four mAbs against DENV2 recombinant NS1 and analyzed their reactivity with the dimeric-NS1 form. The mAbs were also reactive with the native NS1 produced in infected cells but showed different features. The epitope sequences recognized by different mAbs have been recently described and been considered the strategic point for understanding these interactions [15,16,17].

The mAbs 2H5 and 4H1BC showed a similar recognition pattern and share the same epitope binding, ^193^AVHADMGYWIESALNDT^209^. This epitope has been reported as one of the immunodominant B cell epitopes in DENV2 NS1 [20]. This sequence was described in silico and is buried in a beta-sheet structure [15,17]. The recognition of the heat-denatured rNS1 was lower for these mAbs when compared with the non-denatured rNS1, suggesting that these mAbs recognize mainly a conformational epitope. Indeed, by immunofluorescence, both 2H5 and 4H1BC mAbs reacted with dengue virus serotype 2 infected Vero cells. However, they cross-reacted with native ZIKV NS1 in Vero infected cells. The in silico analyses of the similarity of this peptide sequence between different flaviviruses showed that this epitope is highly conserved in these virus but not Yellow fever, Japanese encephalitis and West Nile viruses (Table S2).

The mAb 4F6 recognizes the DENV2 complex-conserved LD2 epitope ^25^VHTWTEQYKFQPES^38^, located on the surface of NS1 fusion loop [8,15,17]. A previous study showed a mAb that binds in this motif is able to recognize the purified NS1 hexamer from all four DENV serotypes [21]. However, 4F6 mAb was able to detect only DENV2 native monomeric NS1 by immunofluorescence but no other serotypes. The divergent results may be accounted to methodological issues, since immunofluorescence is less sensitive and aims to detect infected cells expressing mainly monomeric intracellular NS1, while the purified hexamers were used in an ELISA-based detection system. Hence, this epitope may be exposed depending on the NS1 oligomeric level and the DENV serotype.

The fourth mAb obtained, 4H2, recognized the amino acid sequence ^127^ELHNQTFLIDGPETAEC^143^. A preceding work described a shorter sequence, ^125^STESHNQTFL^134^ exposed in the same loop of DENV2 NS1 [15]. Interestingly, the sequence herein described has nine additional amino acids not previously reported as a B cell epitope and shifting the exposed region to a beta-sheet structure. It recognizes the native protein assessed by immunofluorescence of the four DENV serotypes infected Vero cells, but it did not cross-react with native ZIKV NS1 in Vero-infected cells.

Differentiation of DENV and ZIKV infections is a challenge for current serological tests, particularly in areas where both viruses circulate and co-infection can occur. Thus, mAbs, like 4H2, may be particularly useful for the development of an immunofluorescence based-assay that minimizes the risks associated with false positive results among ZIKV-infected subjects.

## 4. Materials and Methods

### 4.1. Viral Strains and Viral Antigen

The obtention of purified DENV2 NS1 dimers was achieved after denaturation/refolding steps of the protein expressed in *E. coli* followed by affinity chromatography, as previously reported [22]. This recombinant protein was utilized as an antigen for monoclonal antibody development and characterization. Four dengue serotypes and one Zika virus strain were used for further characterization of the mAbs obtained: a dengue virus serotype 2 JHA1 strain [23,24], a rDEN1Δ30 vaccine strain obtained by Δ30 deletion in 3′UTR of DENV1 Western Pacific strain [25], a rDEN3Δ30/31-7164 vaccine strain obtained by Δ30 and Δ31 deletions in 3′ untranslated region (UTR) of DENV3 Slemann/78 strain [26], a rDEN4Δ30 vaccine strain obtained by Δ30 deletion in 3′ UTR of DENV4 Dominica/81 strain [27], and a Brazilian Zika virus strain (ZIKVBR) (Evandro Chagas Institute, Belem, PA, Brazil).

### 4.2. Dengue NS1 Monoclonal Antibody (mAb) Production

Four to six week-old female BALB/c mice were immunized via footpad route with 10 μg rNS1 adsorbed to 1 μg recombinant heat-labile toxin (rLT) [22] as adjuvant. The immunization protocols consisted of three booster injections of the rNS1 and rLT in 0.01 M phosphate buffered saline (PBS), pH 7.4 at 15 days intervals. The mouse with the highest antibody titer was boosted with 10 μg of rNS1 three days prior to cell fusion. The popliteal lymph node cells were fused to SP2/O-Ag14 mouse myeloma cells (2:1) using polyethylene glycol 1500 (Sigma Aldrich, St Louis, MO, USA) [28], with modifications [29]. The supernatant fluids were screened for specific antibodies by indirect ELISA in which 100 μL of hybridoma supernatant was added to a 96-well MaxiSorp microplates (Nunc^®^, Rochester, NY, USA) previously coated with 1 μg/mL of purified rNS1 to screen cultures for antibody production. Antibody-secreting cells were expanded and cloned at limiting dilution [29]. This study was carried out in accordance with the recommendations of Ethical Principles in Animal Research, adopted by the Brazilian College of Animal Experimentation. The protocol was approved by the Ethical Committee for Animal Research of Butantan Institute (995/12).

### 4.3. Dengue NS1 mAbs Characterization

Hybridoma supernatants were incubated with each of the anti-isotype (anti-IgG1, anti-IgG2a, anti-IgG2b, anti-IgG3, anti-IgA and anti-IgM antibodies) previously coated at MaxiSorp microplates followed by incubation with horseradish peroxidase-conjugated rabbit anti-mouse-IgG+A+M+ (1:1000) (Zymed, San Francisco, CA, USA) [27]. The supernatants from selected clones were filtered (0.45 μm) and purified by protein G affinity chromatography (GE-Healthcare, Freiburg, Germany). MAb purity was observed in a 12% polyacrylamide gel electrophoresis containing sodium dodecyl sulphate (SDS-PAGE) staining with Coomassie blue R-250.

The detection limit was established using rNS1 concentrations from 1 to 512 ng coated on microplates, followed incubation with 10 μg/mL of NS1 mAb and with goat anti-mouse peroxidase-conjugated antibody (Invitrogen, Carlsbad, CA, USA) diluted 1:5000. The three-step ELISA was employed to determine the dissociation constants (KD) of antigen-antibody interactions under equilibrium conditions [28].

ELISA assay was also applied in order to observe the reactivity of mAb NS1 against intact and denatured rNS1. For this, MaxiSorp microplates (Thermo Fischer Scientific, Waltham, MA, USA) were coated with 4 μg/mL of Dengue virus serotype 2 (DENV2) rNS1 heat-treated (100 °C for 10 min) or non-heated. The NS1 mAbs were serially diluted (log2) in an initial concentration of 2.5 µg/mL followed by incubation with goat anti-IgG mouse conjugated with horseradish peroxidase (1:10,000).

The reactivity of NS1 mAb against intact and denatured rNS1 was also analyzed by immunoblotting. Thus, 1 µg or 0.5 µg of rNS1 denatured (heat-treated for 10 min at 100 °C) or intact (non-heated) were separated by electrophoresis in denaturing condition polyacrylamide gel containing sodium dodecyl sulphate (SDS-PAGE) 15%. Nitrocellulose membranes (GE-Healthcare, Freiburg, Germany) containing the transferred proteins were tested with NS1 mAbs at a final concentration of 200 ng/mL. Thus, the membranes were incubated with goat anti-IgG mouse conjugated with peroxidase (1:10,000). The reactive protein bands were identified by exposing membranes to a solution of luminol-hydrogen peroxide according to the manufacturer’s instructions (Sigma Aldrich, St Louis, MO, USA). Images were captured by Image Lab™ software (Bio-Rad, Hercules, CA, USA).

### 4.4. Epitope Characterization and Structure Analysis

Peptide mapping was performed using CelluSpot Peptide Array (Intavis, Heidelberg, Germany) following the manufacturer’s recommendations. The slides were produced with dots containing 11 amino acids with overlapping of eight amino acids. Briefly, the slides were blocked, followed by incubation with 30 μg/mL mAb. Next, the slides were incubated with anti-mouse horseradish peroxidase conjugate (1:5000). After washing, diaminobenzidine and hydrogen peroxide were added and the reaction was stopped by the addition of distilled water.

We employed PyMol program (DeLano Scientific LLC, San Carlos, CA, USA, 2009) to predict the structure and the epitope of NS1. For the NS1 structure, we used the available PDB file from Protein Data Bank (code: 4O6B) [6]. For the structure of monoclonal antibodies, we first performed the prediction with Phyre [30].

### 4.5. NS1 Sequences Database Building

One database consisting of amino acid sequences of the NS1 protein in FASTA format was built. Sequences were retrieved from the National Center for Biotechnology Information (NCBI) protein database. Sequences from serotype 1 of DENV have the following accession numbers: ABG75766, ABG75761 and AFN54943. Sequences from serotype 2 of DENV have the following accession numbers: AIE17400, ABK51383 and AFZ40226. Sequences from serotype 3 of DENV have the following accession numbers: ADM63678, AAT79552 and ALI16137. Sequences from serotype 4 of DENV have the following accession numbers: AGI95993, ALB78116 and AFD53008. Sequences from ZIKV isolates have the following accession numbers: AMR39836, AMD61710, ASK51714, ARB07991, AMD16557 and ARB07967.

### 4.6. Conservancy Analysis

The IEDB conservancy analysis was used to determine the conservancy of epitopes for monoclonal antibodies 4F6, 4H2, 2H5 and 4H1BC. A sequence identity threshold of ≥20% was applied.

### 4.7. NS1 mAbs Reactivity to Dengue Virus Serotypes and Zika Virus

Vero cells grown on six-well plates were infected with the viral strains at a MOI of 0.5 for 48 h for DENV and at a MOI of 0.05 for 72 h for ZIKV. The cells were fixed with 1% formaldehyde for 10 min at 4 °C and then permeabilized with 0.5% saponin in PBS for DENV and with cold acetone at −20 °C for ZIKV. The cells were then blocked with PBS containing 10% bovine fetal serum for 30 min. Both cells were treated with mAbs diluted in permeabilization buffer at a concentration of 10 µg/mL for 1 h at room temperature for DENV and 37 °C for 30 min for ZIKV. After three washing steps with PBS, the cells were further treated with Alexa fluor488^®^ (Thermo Fisher, Waltham, MA, USA) conjugated goat anti-mouse IgG at room temperature for 1 h. After another washing period (five times) with PBS, cells were examined using an EVOS digital inverted microscope. Mock infected and cell infected marked with just secondary antibody was the negative control. Also, Vero cells infected with ZIKVBR were tested in order to determine the cross reactivity of the mAbs.

## 5. Conclusions

In the present study we generated four monoclonal antibodies against the nonstructural protein 1 (NS1) of dengue virus serotype 2. One of them (4H2 mAb) recognizes by immunofluorescence the four-dengue virus serotype and did not cross react to zika virus. Thus, the selection and characterization of the epitope, specificity of anti-NS1 mAbs, may contribute to the development of diagnostic tools able to differentiate DENV and ZIKV infections.

## Figures and Tables

**Table 1:** Characteristics of the monoclonal antibodies (mAbs) against dengue virus (DENV) nonstructural protein 1 (NS1).

Name	4F6	4H2	2H5	4H1BC
IgG Subtype ^a^	IgG2a	IgG2a	IgG1	IgG1
DENV2 NS1 reactivity ^b^	Yes	Yes	Yes	Yes
Dissociation Constant (KD) ^c^	1.1 × 10^−8^ M	6.2 × 10^−8^ M	7.3 × 10^−8^ M	8.4 × 10^−8^ M
Detection limit ^d^	16 ng/mL	32 ng/mL	32 ng/mL	32 ng/mL
Epitope sequence ^e^	^25^VHTWTEQYKFQPES^38^	^127^ELHNQTFLIDGPETAEC^143^	^193^AVHADMGYWIESALNDT^209^	^193^AVHADMGYWIESALNDT^209^
DENV (1–4) reactivity ^f^	No	Yes	No	No
ZIKV reactivity ^g^	No	No	Yes	Yes

^a^ The Ig isotype and IgG subtypes were performed by enzyme-linked immunosorbent assay (ELISA) using anti-IgA, anti-IgM, anti-IgG1, anti-IgG2a, anti-IgG2b and anti-IgG3 coated onto microplates; ^b^ The Dengue virus serotype 2 (DENV2) NS1 reactivity was evaluated by indirect ELISA and immunoblotting using rNS1; ^c^ Dissociation constant was performed by ELISA [18]; ^d^ Detection limit was evaluated by ELISA using different concentrations of rNS1; ^e^ The conservancy of DENV2 NS1 epitopes recognized by specific mAbs in a peptide array was analyzed among the four serotypes of DENV, using three samples of NS1 amino acid sequences as representative of each DENV serotype; ^f,g^ DENV (1–4) and Zika virus (ZIKV) reactivity was evaluated by immunofluorescence in Vero cells infected with the specific virus strains.

## Supplementary Materials

The following are available online at http://www.mdpi.com/2073-4468/6/4/14/s1, Figure S1: Characterization of NS1-specific mAbs reactivity by immunoblotting; Figure S2: Epitope mapping with NS1-derived synthetic peptides; Table S1: Epitope analysis of conservancy in DENV serotypes. Table S2: Epitope analysis of conservancy in different strains of DENV, ZIKV, YFV, JEV and WNV strains.
