# Supplementary material for: Epitope Sequences in Dengue Virus NS1 Protein Identified by Monoclonal Antibodies
Source: Antibodies (Basel). 2017 Oct 15;6(4):14. doi: 10.3390/antib6040014 (PMC6698852; doi:10.3390/antib6040014)
Supplement: Supplementary file 1 [file antibodies-06-00014-s001.pdf]

Supplementary Materials: Epitope Sequences in Dengue Virus NS1 Protein Identified by Monoclonal Antibodies

Leticia Barboza Rocha [1,†], Rubens Prince dos Santos Alves [2,†], Bruna Alves Caetano [1], Lennon Ramos Pereira [2], Thais Mitsunari [1], Jaime Henrique Amorim [2,‡], Juliana Moutinho Polatto [1], Viviane Fongaro Botosso [3], Neuza Maria Frazatti Gallina [4], Ricardo Palacios [5], Alexander Roberto Precioso [5], Celso Francisco Hernandes Granato [6], Danielle Bruna Leal Oliveira [7], Vanessa Barbosa da Silveira [7], Daniela Luz [1], Luís Carlos de Souza Ferreira [2] and Roxane Maria Fontes Piazza [1,*]

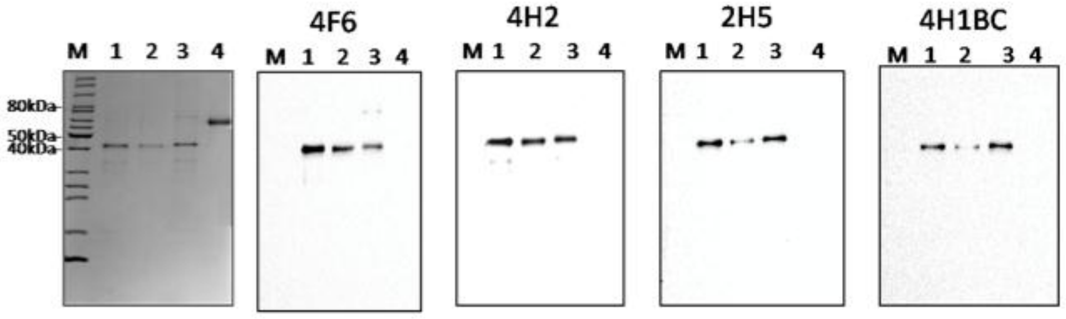

Figure S1. Characterization of NS1-specific mAbs reactivity by immunoblotting.

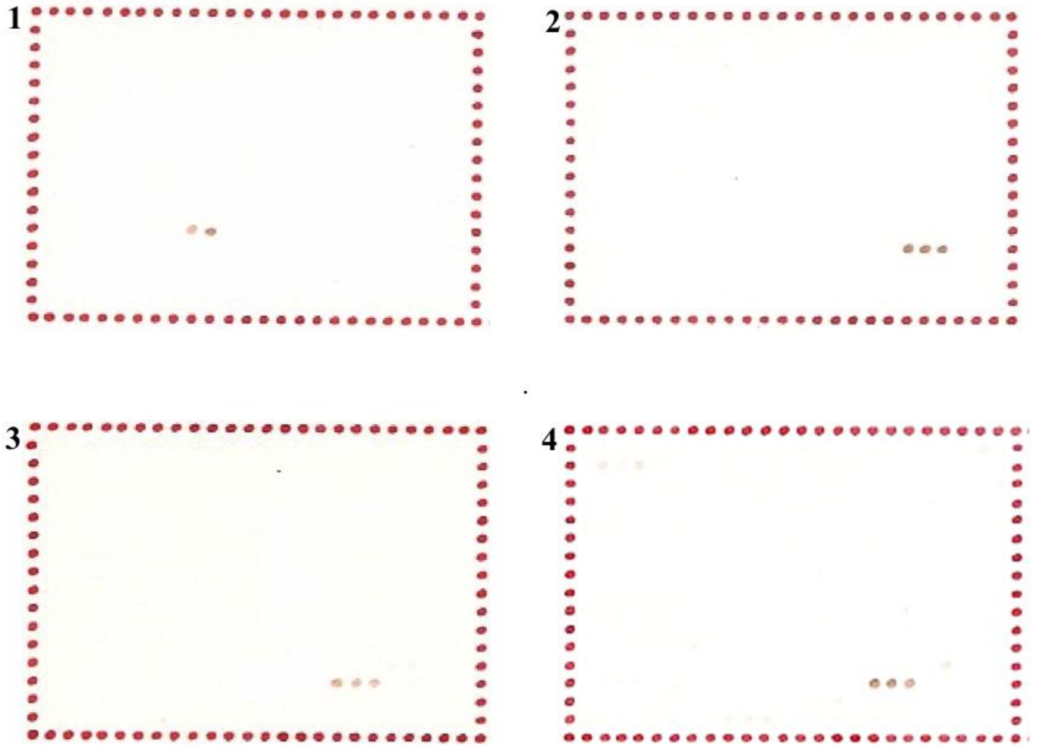

Figure S2. Epitope mapping with NS1-derived synthetic peptides.

Table S1. Epitope analysis of conservancy in different strains of DENV serotypes and Zika strains.

Protein Name	Positions	Protein Sub–Sequence(s)	Identity
	**4F6 mAb**		
DENV1ns1_AAN06982USA	25–38	VHTWTEQYKFQADS	85.71%
DENV1ns1_ABK27927China	25–38	VHTWTEQYKFQADS	85.71%
DENV2_JHA1	25–38	VHTWTEQYKFQPES	100.00%
DENV2_NGC	25–38	VHTWTEQYKFQPES	100.00%
DENV2_DENV2_FJ687441_Thailand	25–38	VHTWTEQYKFQPES	100.00%
DENV3ns1_AAS49486	23–36<br>401–414	VHTWTEQYKFQADS<br>VHTWTEQYKFQADS	85.71%
DENV3ns1_AAX19004Indonésia	25–38	VHTWTEQYKFQADS	85.71%
DENV4ns1_AAK01233USA	25–38	VHTWTEQYKFQPES	100.00%
DENV4ns1_Q5UCB8	24–37	VHTWTEQYKFQPES	100.00%
DENV4_GQ868585_Colombia	25–38	VHTWTEQYKFQPES	100.00%
AMR39836_Zika_virus	23–36	VEAWRDRYKYHPDS	42.86%
AMD61710_Zika_virus	23–36	VEAWRDRYKYHPDS	42.86%
ASK51714_Zika_virus	25–38	VEAWRDRYKYHPDS	42.86%
ARB07967_Zika_virus	25–38	VEAWRDRYKYHPDS	42.86%
AMD16557_Zika_virus	23–36	VEAWRDRYKYHPDS	42.86%
	**4H2 mAb**		
DENV2_JHA1	127–143	ELHNQTFLIDGPETAEC	100.00%
DENV2_NGC	127–143	ELHNQTFLIDGPETAEC	100.00%
DENV2_DENV2_FJ687441_Thailand	127–143	ESHNQTFLIDGPETAEC	94.12%
DENV4ns1_AAK01233USA	127–143	EARNSTFLIDGPDTSEC	70.59%
DENV4ns1_Q5UCB8	126–142	EARNSTFLIDGPDTSEC	70.59%
DENV4_GQ868585_Colombia	127–143	EARNSTFLIDGPDTSEC	70.59%
AMR39836_Zika_virus	125–141	AKTNNSFVVDGDTLKEC	35.29%
AMD61710_Zika_virus	125–141	AKTNNSFVVDGDTLKEC	35.29%
ASK51714_Zika_virus	127–143	AKTNNSFVVDGDTLKEC	35.29%
ARB07967_Zika_virus	127–143	AKTNNSFVVDGDTLKEC	35.29%
AMD16557_Zika_virus	125–141	AKTNNSFVVDGDTLKEC	35.29%
	**2H5 mAb**		
DENV1_GU131863_SãoPaulo (Brazil)	193–209	AVHADMGYWIESEKNET	82.35%
DENV1ns1_AAN06982USA	193–209	AVHADMGYWIESEKNET	82.35%
DENV1ns1_ABK27927China	193–209	AVHADMGYWIESEKNET	82.35%
DENV2_JHA1	193–209	AVHADMGYWIESALNDT	100.00%
DENV2_NGC	193–209	AVHADMGYWIESALNDT	100.00%
DENV2_DENV2_FJ687441_Thailand	193–209	AVHADMGYWIESALNDT	100.00%
DENV3ns1_AAS49486	191–207<br>569–585	AVHADMGYWIESQKNGS,<br>AVHADMGYWIESQKNGS	76.47%
DENV3ns1_AAX19004Indonésia	193–209	AVHADMGYWIESQKNGS	76.47%
DENV4ns1_AAK01233USA	193–209	AVHADMGYWIESSKNQT	82.35%
DENV4ns1_Q5UCB8	192–208	AVHADMGYWIESSKNQT	82.35%

DENV4_GQ868585_Colombia	193–209	AVHADMGYWIESSKNQT	82.35%
AMR39836_Zika_virus	191–207	AAHSDLGYWIESEKNDT	70.59%
AMD61710_Zika_virus	191–207	AVHSDLGYWIESEKNDT	76.47%
ASK51714_Zika_virus	193–209	AVHSDLGYWIESEKNDT	76.47%
ARB07967_Zika_virus	193–209	AVHSDLGYWIESEKNDT	76.47%
AMD16557_Zika_virus	191–207	AVHSDLGYWIESEKNDT	76.47%
**4H1BC mAb**			
DENV1_GU131863_SãoPaulo(Brazil)	193–209	AVHADMGYWIESEKNET	82.35%
DENV1ns1_AAN06982USA	193–209	AVHADMGYWIESEKNET	82.35%
DENV1ns1_ABK27927China	193–209	AVHADMGYWIESEKNET	82.35%
DENV2_JHA1	193–209	AVHADMGYWIESALNDT	100.00%
DENV2_NGC	193–209	AVHADMGYWIESALNDT	100.00%
DENV2_DENV2_FJ687441_Thailand	193–209	AVHADMGYWIESALNDT	100.00%
DENV3ns1_AAS49486	191–207<br>569–585	AVHADMGYWIESQKNGS<br>AVHADMGYWIESQKNGS	76.47%
DENV3ns1_AAX19004Indonésia	193–209	AVHADMGYWIESQKNGS	76.47%
DENV4ns1_AAK01233USA	193–209	AVHADMGYWIESSKNQT	82.35%
DENV4ns1_Q5UCB8	192–208	AVHADMGYWIESSKNQT	82.35%
DENV4_GQ868585_Colombia	193–209	AVHADMGYWIESSKNQT	82.35%
AMR39836_Zika_virus	191–207	AAHSDLGYWIESEKNDT	70.59%
AMD61710_Zika_virus	191–207	AVHSDLGYWIESEKNDT	76.47%
ASK51714_Zika_virus	193–209	AVHSDLGYWIESEKNDT	76.47%
ARB07967_Zika_virus	193–209	AVHSDLGYWIESEKNDT	76.47%
AMD16557_Zika_virus	191–207	AVHSDLGYWIESEKNDT	76.47%

Table S2. Epitope analysis of conservancy in different strains of DENV, ZIKV, YFV, JEV and WNV strains.

2H5 and 4H1BC mAb			
Protein name	Positions	Protein sub-sequence(s)	Identity
ABG75766_Dengue_virus_1	192-208	AVHADMGYWIESEKNET	82.35%
ABG75761_Dengue_virus_1	192-208	AVHADMGYWIESEKNET	82.35%
AFN54943_Dengue_virus_1	192-208	AVHADMGYWIESEKNET	82.35%
AIE17400_Dengue_virus_2	192-208	AVHADMGYWIESALNDT	100.00%
ABK51383_Dengue_virus_2	192-208	AVHADMGYWIESALNDT	100.00%
AFZ40226_Dengue_virus_2	192-208	AVHADMGYWIESALNDT	100.00%
ADM63678_Dengue_virus_3	191-207	AVHADMGYWIESQKNGS	76.47%
AAT79552_Dengue_virus_3	191-207	AVHADMGYWIESQKNGS	76.47%
ALI16137_Dengue_virus_3	191-207	AVHADMGYWIESQKNGS	76.47%
AGI95993_Dengue_virus_4	191-207	AVHADMGYWIESSKNQT	82.35%
ALB78116_Dengue_virus_4	192-208	AVHADMGYWIESSKNQT	82.35%
AFD53008_Dengue_virus_4	191-207	AVHADMGYWIESSKNQT	82.35%
AMR39836_Zika_virus	191-207	AAHSDLGYWIESEKNDT	70.59%
AMD61710_Zika_virus	191-207	AVHSDLGYWIESEKNDT	76.47%
ASK51714_Zika_virus	193-209	AVHSDLGYWIESEKNDT	76.47%
ARB07991_Zika_virus	193-209	AVHSDLGYWIESEKNDT	76.47%
ARB07978_Zika_virus	193-209	AVHSDLGYWIESEKNDT	76.47%
AMD16557_Zika_virus	191-207	AVHSDLGYWIESEKNDT	76.47%
ARQ19026_Yellow_fever_virus	171-187,203-219	AVFEYTMDCDGSILGAA, GSHEVNGTWMIHTLETL	23.53%
ARM37843_Yellow_fever_virus	171-187,203-219	AVFEYTMDCDGSILGAA, GSHEVNGTWMIHTLETL	23.53%
ARM37842_Yellow_fever_virus	171-187,203-219	AVFEYTMDCDGSILGAA, GSHEVNGTWMIHTLETL	23.53%
AAA21436_Japanese_encephalitis_virus	193-209	AVHSDLSYWIESRYNDT	70.59%
AAA81554_Japanese_encephalitis_virus	193-209	AVHSDLSYWIESRYNDT	70.59%
ADT63077_Japanese_encephalitis_virus	193-209	AVHSDLSYWIESRFNDT	70.59%
ABC49716_West_Nile_virus	193-209	AVHSDLSYWIESGLNDT	76.47%
AIY22515_West_Nile_virus	193-209	AIHSDLSYWIESRLNDT	70.59%
AFX61606_West_Nile_virus	193-209	AIHSDLSYWIESRLNDT	70.59%
